# Cancer-Cell Deep-Learning Classification by Integrating Quantitative-Phase Spatial and Temporal Fluctuations

**DOI:** 10.3390/cells10123353

**Published:** 2021-11-29

**Authors:** Shani Ben Baruch, Noa Rotman-Nativ, Alon Baram, Hayit Greenspan, Natan T. Shaked

**Affiliations:** Department of Biomedical Engineering, Tel Aviv University, Tel Aviv 6997801, Israel; shani3@mail.tau.ac.il (S.B.B.); noale.nativ@gmail.com (N.R.-N.); alontbst@gmail.com (A.B.); hayit@eng.tau.ac.il (H.G.)

**Keywords:** cancer cells, classification, fluctuations, deep learning

## Abstract

We present a new classification approach for live cells, integrating together the spatial and temporal fluctuation maps and the quantitative optical thickness map of the cell, as acquired by common-path quantitative-phase dynamic imaging and processed with a deep-learning framework. We demonstrate this approach by classifying between two types of cancer cell lines of different metastatic potential originating from the same patient. It is based on the fact that both the cancer-cell morphology and its mechanical properties, as indicated by the cell temporal and spatial fluctuations, change over the disease progression. We tested different fusion methods for inputting both the morphological optical thickness maps and the coinciding spatio-temporal fluctuation maps of the cells to the classifying network framework. We show that the proposed integrated triple-path deep-learning architecture improves over deep-learning classification that is based only on the cell morphological evaluation via its quantitative optical thickness map, demonstrating the benefit in the acquisition of the cells over time and in extracting their spatio-temporal fluctuation maps, to be used as an input to the classifying deep neural network.

## 1. Introduction

The classification of cancerous cells is an important medical endeavor of great significance for biophysical assays and medical diagnoses. Attempts to compare healthy cells to cancerous cells have been made for years, and numerous biomarkers have been discovered to distinguish between them. Most of these biomarkers are based on using exogenous cell labeling, which requires prior knowledge of the labeling agent. Alternatively, label-free measurements of the intrinsic properties of the cells for cell discrimination can be used. One of the important label-free biomarkers is cell stiffness, which is associated with disease invasiveness [1,2,3,4,5,6]. Most studies have shown that metastatic cancer cells, which have been detached from the tumor, are less stiff and have a higher migratory and invasive potential than primary tumor cells.

A popular method for determining cell stiffness is by using atomic force microscopy (AFM) [3,4,5,6]. However, this approach is complicated, slow, expensive, and difficult to execute in clinics. Other techniques, such as micropipette aspiration, optical tweezers, and magnetic twisting cytometry [7,8], also show a substantial inverse correlation between cancer-cell stiffness and metastatic potential. However, these methods are cell invasive and may lead to cell damage.

Non-invasive techniques, such as interferometric phase microscopy (IPM), can record holographic images of biological cells. These images provide the dynamic optical path delay (OPD) topographic maps of the cells without the use of cell staining [9,10,11]. We have previously shown that various morphological and textural parameters derived directly from the cell OPD maps, including the cell dry mass and phase volume, can be used to obtain statistical differences between healthy and different cancer cells [12]. The OPD temporal fluctuations can be used as an indicative analysis tool (e.g., [13,14]). Specifically, the mean standard deviation of the cell temporal fluctuations, as extracted from the cell OPD profile over time, can be used to obtain statistical differences between healthy and different cancer cells [15]. The cell fluctuations are indicative of the cell stiffness, where cancer cells change their stiffness as cancer progresses, in addition to their three-dimensional (3D) morphology. Furthermore, the OPD map can be used to calculate the cell spatial fluctuations, which are the basis for the cell disorder strength, a parameter that is also linked to cell stiffness [16,17]. Thus, both the morphology and the spatio-temporal fluctuations should be taken into account when evaluating the cell metastatic potential.

Previous studies have used simple machine-learning classifiers on cell OPD maps to discriminate between various cellular conditions (e.g., [12,18]). With advances in deep learning, other studies have distinguished between the invasiveness levels of cancer cells. Most of them used only the OPD-based morphology of the cell (e.g., [19,20]). Deep-learning classifiers based on temporal fluctuations have been previously used to classify red blood cells [21] and SARS-CoV-2 virus particles [22].

Here we present a new method that uses the cell temporal fluctuations, on top of the cell spatial fluctuations and morphology, as the basis for cancer-cell classification. This requires capturing a video of the OPD maps of the cells and extracting the temporal and spatial fluctuations as an input for the classifier. The fact that we now have very different domains characterizing the cells, the morphological OPD domain and the spatio-temporal fluctuations domain gives rise to the question of how to fuse them into the classifying network architecture. This is going to be explored as well.

In general, the use of deep learning to classify videos involves methods such as 3D convolutional neural networks (CNN), optical flow computation, and long short-term memory (LSTM) networks [21,23,24,25]. These methods would fail to capture extremely minor changes for hundreds of frames, which is needed for quantifying cancer cell fluctuations. Our approach, on the other hand, extracts both the spatial and temporal fluctuations from the video as one image per cell, and utilizes a unique triple-path neural-network architecture to integrate the cell morphology and cell fluctuations, and use them for cell classification.

## 2. Materials and Methods

### 2.1. Dataset

Our dataset contains 216 videos of live colorectal adenocarcinoma cancer cells. Each video contains a single cell. The primary colon cancer cells (from SW480 cell line) contained 104 videos, whereas the metastatic colon cancer cells (from SW620 cell line) contained 112 videos. Both cell lines were purchased from the ATCC and originated from the same patient. Each video was recorded for 4 s at a rate of 500 frames/sec with a resolution of 512 × 512 pixels.

### 2.2. Cell Preparation and Imaging

**Figure 1:**
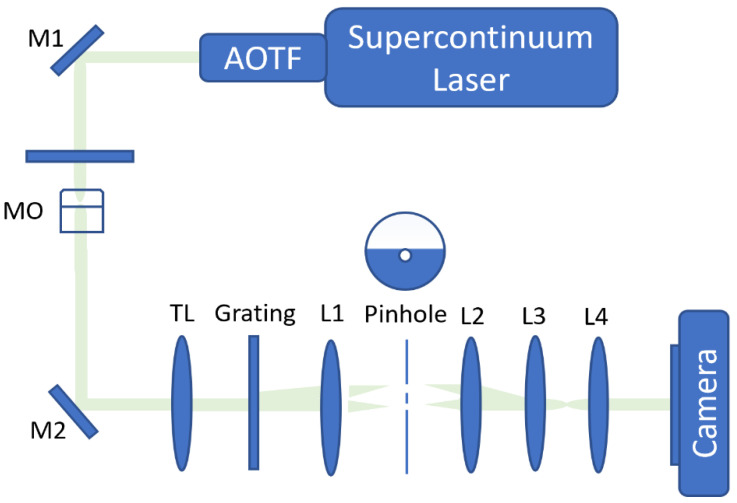
Setup scheme. An inverted microscope with a DPM module positioned at its output. AOTF, acousto-optic tunable filter; M1, M2, mirrors; sample; MO, microscope objective; TL, tube lens; G, amplitude diffraction grating; L1–L4, lenses.

The cells were grown in a Dulbecco’s Modified Eagle Medium (DMEM) until 80% confluence, and then trypsinized to suspend them. The optical system used to acquire the cells is presented in Figure 1. This system is based on diffraction phase microscopy (DPM) [26]. A commercial microscope (IX83, Olympus) was illuminated by a low-coherence laser beam (SuperK Extreme, NKT) connected to an acousto-optical tunable filter, creating a wavelength bandwidth of 633 ± 2.5 nm. The sample passed through the cell sample and was magnified by a 40× microscope objective and projected by a tube lens onto the microscope output plane, where a custom-built DPM module was located. This is an external common-path interferometric module that creates an off-axis hologram on the camera. This is done by using a diffraction grating to split the sample image into two beams and erasing the sample information from one of the beams using spatial filtering, effectively turning it into a reference beam. The spatial filtering is implemented by a 4f lens configuration, and a pinhole located at the confocal plane. Another 4f lens configuration creates a secondary magnification of 6×. The off-axis hologram is captured by a fast camera (Photron FASTCAM Mini AX200).

**Figure 2:**
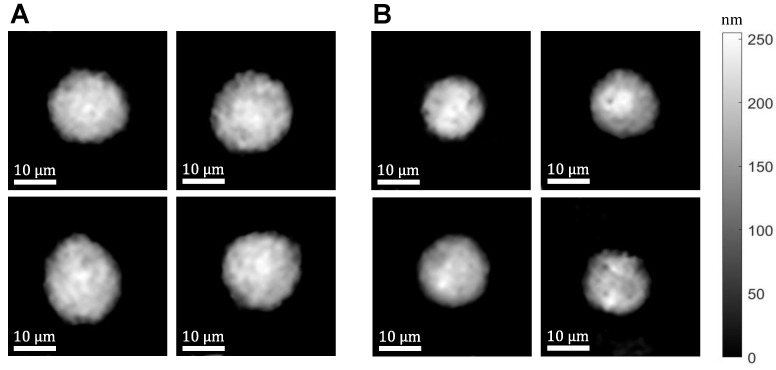
Examples of cell OPD maps from the dataset. (**A**) SW480 cells; (**B**) SW620 cells.

We extracted the optical path delay (OPD) from each of the recorded off-axis holograms using the digital procedure described in [27,28], including a Fourier transform cropping one of the cross-correlation terms, an inverse Fourier transform, two-dimensional phase unwrapping on the phase argument, and a division by 2π/λ, where λ is the central illumination wavelength. Then, each OPD video per cell is resized to 250 × 250 pixels. Examples of the resulting cell OPD maps are presented in Figure 2.

### 2.3. Pre-Processing Technique

Regular approaches for classifying videos in deep learning aim to capture substantial displacement in space, such as throwing a ball. In our case, we have to capture subtle OPD fluctuations in cancer cells, so those methods would fail. In addition, we need to deal with a large amount of data (2000 frames for each cell), while capturing the essence of the movements. 

**Figure 3:**
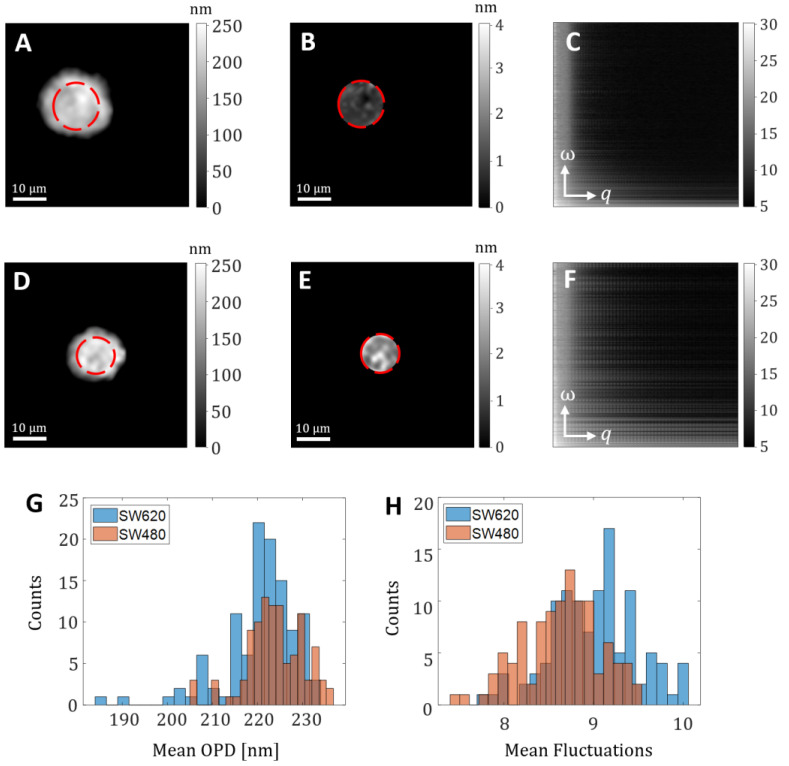
Demonstration of the preprocessing technique. (**A**–**C**) SW480 cell. (**D**–**F**) SW620 cell. (**A**,**D**) OPD map of the cell; (**B**,**E**) Temporal standard deviation (std) of the cell OPD video; (**C**,**F**) Fluctuation map; *ω*, temporal frequency; *q* spatial frequency. Red circles mark the analyzed areas, excluding the cell edges. (**G**) Histogram of the mean OPD value across the cell area for the two groups of cells, with *p* < 0.0005 between the groups. (**H**) Histogram of the mean of the absolute values of the spatio-temporal fluctuation map, with *p* < 0.0005 between the groups.

To solve this issue, and capture both spatial and temporal frequencies from the video in one image per cell, we used the representation suggested by Popescu et al. [29]. First, we excluded the edges of the cell due to increased vibrations in these areas. For this, we created a mask, based on the first image from each video, and applied this mask to the whole video (see Figure 3a,d). Figure 3b,e present the temporal-fluctuation standard deviation maps obtained. In the following step, for each frame from each video representing one cell in the database, we subtracted the average frame, and calculated the Fourier transform in three dimensions. Then, for each temporal frequency, we calculated the radially averaged power spectral density and built a fluctuation map with two axes: the horizontal axis represents spatial frequencies *q*, and the vertical axis represents temporal frequencies *ω* (see Figure 3c,f). Figure 3g shows a histogram of the mean OPD value on the cell area for the two groups of cells, demonstrating that there is a statistical difference between the groups with *p* < 0.0005, but without full seperation. Figure 3h shows a histogram of the mean of the absolute values of the spatio-temporal fluctuation map, demonstrating again that there is a statistical difference between the groups with *p* < 0.0005, but without full seperation. These results support the development of an automatic classifier on a cell-by-cell basis that takes into consideration both the OPD and the spatio-temporal fluctuations.

### 2.4. Framework for Deep-Learning-Based Classification

We selected the ResNet-50 architecture [30], pre-trained on ImageNet [31], to distinguish between primary cells and metastatic cells. Some CNNs may suffer from low diagnostic performance due to the vanishing-gradient and the divergence-gradient problems, which obstruct information transmission from shallow layers to deep layers of the network. ResNet-50 solves this problem by identifying shortcut connections, skipping certain layers while providing great generalization performance with a relatively small number of parameters. Indeed, ResNet-50 has been successfully used for many medical image classification tasks [32]. We therefore selected it for our case as well. 

We trained this network for each modality separately: (1) the cell stationary morphology (OPD) maps, which formed our baseline model; and (2) the spatio-temporal fluctuation maps described in Section 2.3. Each architecture learns from each modality a specific structure, and can thus lead to better classification results by integrating both modalities. Based on this assumption, we created a multimodal network and used early- and late-fusion techniques [33] to combine the features, as described next.

First, we examined the early-fusion technique, which involves integration between the two modalities before being used as an input to the network. We concatenated each modality (the cell morphology map or the spatio-temporal fluctuation map) across the channel dimension and trained a simple ResNet architecture.

**Figure 4:**
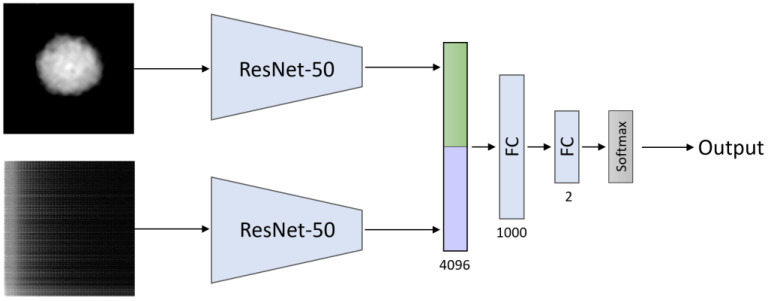
Double-path model architecture. The inputs are the cell OPD morphology map and cell spatio-temporal fluctuation map. We created a feature vector for each modality using a ResNet-50 model. Then, we concatenated the feature vectors and passed the concatenated vector into two fully connected (FC) layers, which gradually decreased the vector size by having a smaller number of outputs than inputs, so that it could finally enter a softmax layer. The numbers below each layer represent the length of the output vector. The output of the overall network is the probability of being a metastatic cancer cell.

Then, we examined the late-fusion technique, which involves the aggregation of decisions from multiple networks, and created a double-path model (see Figure 4). We employed a modified ResNet-50 architecture in each path, removing the last two layers (softmax and fully connected layers) and flattening the output of the average pooling layer. Then, we inserted the cell morphology map or the fluctuation map for each path respectively, thus creating a feature vector for each modality. We used the late-fusion approach and concatenated each feature vector from each modality and passed the unified vector to a classifier.

**Figure 5:**
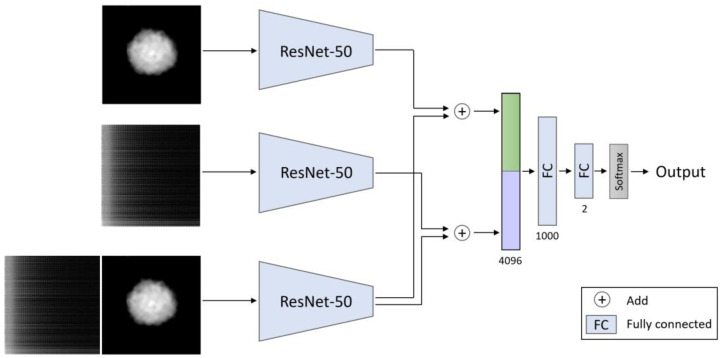
Triple-path model architecture. The input is the cell OPD morphology, cell spatio-temporal fluctuation map, and a concatenation between them. We created a feature vector for each path using a ResNet-50 model. We added the feature vector from the third path to the vector of each of the other two paths. Then, we concatenated between the two resulting vectors. We passed the concatenated vector into two fully connected (FC) layers, which gradually decreased the vector size by having a smaller number of outputs than inputs, so that it could finally enter a softmax layer. The numbers below each layer represent the length of the output vector. The output of the overall network is the probability of being a metastatic cancer cell.

To achieve better performance, we developed a new model with a triple path, as presented in Figure 5. This model combines the advantage of the early-fusion technique, which is the automatic learning of the cross-modal representation, with the advantage of the late-fusion technique, enhancing the features of the different modalities by two independent modules. We employed the same modified ResNet-50 architecture and inserted three inputs: (1) the cell morphology map; (2) the spatio-temporal fluctuation map; (3) a two-channel input that concatenated between the cell morphology and the spatio-temporal fluctuation maps. We extracted a feature vector from each path and added the two-channel feature vector to the cell-morphology feature vector and to the spatio-temporal-fluctuation-map feature vector. Then, we concatenated those two vectors and passed the unified vector to a classifier.

### 2.5. Implementation Details

All models were trained using the cross-entropy (CE) loss with a batch size of 8, a learning rate of 2 × 10^−6^, and an ADAM optimizer. To improve generalization, we randomly created 20 different shuffles. Each shuffle was split into train, validation, and test sets (60%, 20%, 20%), and the epoch with the highest score on the validation set was chosen for the test set. Each shuffle was trained for 100 epochs. The reported results are obtained from the mean and standard deviation (std) across all the shuffles.

## 3. Results

To evaluate the various methods, we used five different measurements: accuracy, sensitivity, specificity, precision, and area under curve (AUC). The results are presented in Table 1. We examined all the different architectures: single-path ResNet, double-path ResNet and triple-path ResNet. We also compared between early and late-fusion techniques by employing different inputs: the morphology, the spatio-temporal fluctuation map and/or the two-channel input, as described in Section 2.4. As presented in Table 1, combining the morphology and spatio-temporal fluctuation map (triple-path model) improves the results from 85% (morphology only) to 89% (combining morphology and spatio-temporal fluctuations).

**Figure 6:**
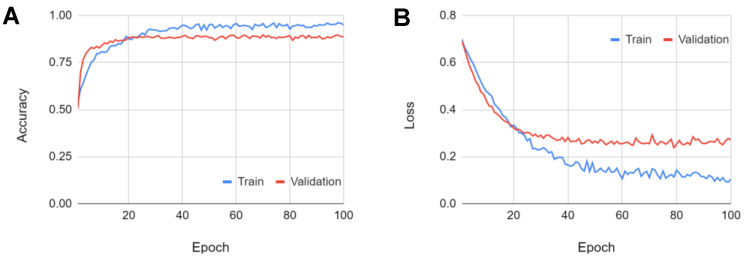
Triple-path model graphs of: (**A**) accuracy, and (**B**) loss, averaged across all shuffles.

Figure 6 shows the shuffle-averaged accuracy and loss of the chosen triple-path model, indicating that no overfitting occurs.

## 4. Discussion and Conclusions

In this work, we presented a new approach for classifying cancer cells based on the cell spatial and temporal fluctuations, in addition to the cell OPD-based morphology. We demonstrated that by using a 3D Fourier transform on the fluctuations, we can extract a single map per cell, quantifying the subtle spatio-temporal fluctuations of the cell. We also demonstrated that by using a smart integration of the OPD-based morphology and fluctuation maps of cancer cells, improved classification performance is obtained, compared to classifying according to the OPD-based morphology alone. Our approach to integration combines various fusion techniques. The early-fusion technique, also known as feature-level fusion, combines the modalities before they are input into the network, resulting in a single feature vector for both modalities. The late-fusion technique, also known as decision-level fusion, first fuses the feature vector for each modality, then merges the vectors and sends the resulting single vector to a classifier. We can see from the results that using both fusion approaches together in the triple-path model yields the best performance.

Note that the cells of the two groups were imaged in several different dishes, prepared together under the same conditions. This procedure minimizes possible differences between dishes. Furthermore, since the collection of each group of cells was done in five different dishes, differences between dishes that might have caused changes between the groups weres further minimized. Thus, the classifier did not learn the difference between the dishes, but between the groups of cells.

Previous approaches [12] used handcrafted features extracted from the cancer cell morphological OPD map to train a simple classifier instead of using the cell OPD map directly, without feature extraction, to train a deep-learning classifier. When classifying between SW420 and SW620 cells, the same cells as analyzed in the current paper, 82% sensitivity, 81% specificity, and 89.7% AUC were obtained. Note that the results presented in Table 1 are better, even for the case of the OPD morphology map representation alone, with 82.38% sensitivity, 88.17% specificity, and 93.66% AUC. Even stronger results are achieved when analyzing the cells based on both the OPD morphology map and the spatio-temporal fluctuation map by the proposed triple-path model, yielding 88.89% sensitivity, 89.32% specificity, and 96.03% AUC. These results demonstrate the usefulness of the proposed deep-learning framework that classifies the cell OPD map and the fluctuation map at the pixel level, and does not need to rely on extracting specific handcrafted features prior to the cell classification task.

With the introduction of portable and easy-to-use interferometric modules [34,35], which are suitable for direct clinical use, the proposed cancer-cell classification method can be utilized for cancer diagnosis in medical clinics, for detection and monitoring of circulating tumor cells isolated from liquid biopsies [36,37], such as blood, as well as for analyzing cancer cells extracted from solid tumors and tissue biopsies. For these clinical applications, correlating the cell metastatic potential with its OPD morphology and spatio-temporal fluctuation maps is of utmost importance, since it can help in diagnosing and monitoring the patient’s disease stage, and define the suitable therapeutic paths. Our method also forms a general analysis and classification approach for red blood cells, as well as other types of cells expressing stiffness changes.

## Figures and Tables

**Table 1:** Performance of the different methods. The values represent the means and the standard deviations of the various shuffles. Bold text mark the best results.

Model	Input	Accuracy %	Sensitivity %	Specificity %	Precision %	AUC %
Single	Morphology	85.35 ± 4.88	82.38 ± 9.11	88.17 ± 8.20	82.82 ± 8.34	93.66 ± 2.72
	Fluctuations	77.09 ± 7.03	70.02 ± 12.74	84.88 ± 7.33	73.12 ± 10.38	88.28 ± 5.29
	2 Channels	86.28 ± 4.87	85.59 ± 7.58	86.67 ± 8.26	85.03 ± 7.24	94.09 ± 3.03
Double	Morphology + Fluc.	85.23 ± 4.11	82.99 ± 9.01	86.67 ± 7.77	83.52 ± 9.06	93.49 ± 2.99
	Morphology + 2 Ch.	87.67 ± 5.36	85.74 ± 7.83	89.58 ± 7.96	85.60 ± 8.96	95.64 ± 2.46
Triple	Morphology + Fluc. + 2 Channels	89.07 ± 4.23	88.89 ± 7.50	89.32 ± 5.80	88.26 ± 7.81	96.03 ± 2.44

## Data Availability

Additional data can be obtained upon a reasonable request.
